# Antibody humanization—the Influence of the antibody framework on the CDR-H3 loop ensemble in solution

**DOI:** 10.1093/protein/gzaa004

**Published:** 2020-03-04

**Authors:** Monica L Fernández-Quintero, Martin C Heiss, Klaus R Liedl

**Affiliations:** Institute of General, Inorganic and Theoretical Chemistry, Center for Molecular Biosciences Innsbruck, University of Innsbruck, Innrain 80-82, A-6020 Innsbruck, Austria

**Keywords:** CDR-H3 loop dynamics, conformational selection, humanization, immunogenicity, interdomain dynamics

## Abstract

Antibody engineering of non-human antibodies has focused on reducing immunogenicity by humanization, being a major limitation in developing monoclonal antibodies. We analyzed four series of antibody binding fragments (Fabs) and a variable fragment (Fv) with structural information in different stages of humanization to investigate the influence of the framework, point mutations and specificity on the complementarity determining region (CDR)-H3 loop dynamics. We also studied a Fv without structural information of the anti-idiotypic antibody Ab2/3H6, because it completely lost its binding affinity upon superhumanization, as an example of a failed humanization. Enhanced sampling techniques in combination with molecular dynamics simulations allow to access micro- to milli-second timescales of the CDR-H3 loop dynamics and reveal kinetic and thermodynamic changes involved in the process of humanization. In most cases, we observe a reduced conformational diversity of the CDR-H3 loop when grafted on a human framework and find a conformational shift of the dominant CDR-H3 loop conformation in solution. A shallow side minimum of the conformational CDR-H3 loop ensemble attached to the murine framework becomes the dominant conformation in solution influenced by the human framework. Additionally, we observe in the case of the failed humanization that the potentially binding competent murine CDR-H3 loop ensemble in solution shows nearly no kinetical or structural overlap with the superhumanized variant, thus explaining the loss of binding.

## Introduction

Antibodies have become one of the most important and fastest growing classes of biotherapeutic proteins (Grilo and Mantalaris, 2019; Carter, 2006, 2011). Long half-life, specificity to their respective antigen and efficacy are obvious benefits of antibodies (Chames *et al*., 2009). However, the major challenge in developing therapeutics still lies in overcoming the tendency to show immunogenic responses to non-human derived antibodies (Borrebaeck, 1997). Non-human derived antibodies like murine antibodies, because of their foreign characteristics, especially foreign sequences, can lead to a human anti-mouse antibody (HAMA) response (Hwang and Foote, 2005). HAMA responses have motivated ongoing improvements in addressing the immunogenicity risk by reducing the murine content, starting with chimeric and humanized monoclonal antibodies (mAbs) until the development of fully human mAbs (Chames *et al*., 2009). The chimeric mAb is based on fusing the murine variable domains with the human constant domains, while humanization describes the grafting of only the murine antibody complementarity determining region (CDR) onto a human germline framework. Still the treatment with humanized antibodies can trigger human anti-human responses (Nechansky, 2010). Therefore, the challenge in the humanization process is to maintain the full biological function, reflected in a high binding affinity and to substantially reduce the risk of adverse side-effects (Shankar *et al*., 2006). Various advanced protocols focused on refining the humanization strategies by resurfacing the mAb, comparing the solvent accessibility of human and murine antibodies (Roguska *et al*., 1994), superhumanization (Tan *et al*., 2002) and immunizing transgenic mice (Brüggemann *et al*., 2015) and specific human content optimization (Lazar *et al*., 2007). Characterization of the antigen-binding site (Nguyen *et al*., 2017) (paratope) and antibody binding properties (Ma *et al*., 1999) is crucial for understanding the function of the antibody. The most important region involved in the antigen-binding process is the CDR consisting of six hypervariable loops that shape the paratope (Chothia and Lesk, 1987; Al-Lazikani *et al*., 1997; Nowak *et al*., 2016; Daisuke *et al*., 2009). Mainly the CDR loops of the heavy chain (Xu and Davis, 2000) are described to be involved in antigen-binding, especially the CDR-H3 loop (Regep *et al*., 2017). The CDR-H3 loop is known to play a central role in antigen recognition and has on average the highest counts of contacts with antigens (Tsuchiya and Mizuguchi, 2016; MacCallum *et al*., 1996; Edelman, 1973). The backbone conformations of the CDR loops except the CDR-H3 loop have been classified into canonical structures according to their loop length and sequence composition (Chothia and Lesk, 1987; North *et al*., 2011). The CDR-H3 loop, due to its high diversity in length, sequence and structure and its ability to adopt various different conformations during the V(D)J recombination and somatic hyper-mutation, remains challenging to be predicted accurately (Regep *et al*., 2017; Tonegawa, 1983; Hozumi and Tonegawa, 1976; Wabl and Steinberg, 1996). CDR-H3 loop length and structure can have an effect on the antigen-binding patterns of the other CDR loops and influence the specificity of the paratope for target antigens (Tsuchiya and Mizuguchi, 2016). However, especially the antibody humanization process revealed the substantial role of the framework on the CDR loop binding properties (Bernardi *et al*., 1850). We analyzed five different antibody humanization series with structural information in different stages of humanization to identify the influence of the framework on the CDR-H3 loop dynamics. The first antibody shows the humanization of a chimeric anti-human IL-13 antibody Fab using the human framework adaptation method. This method does not only contain the human framework selection but consists of a specificity-determining residue optimization (Fransson *et al*., 2010). IL-13 is a member of the growth-hormone-like cytokine family and plays a central role in the development of asthma (Grünig *et al*., 2012). The second antibody describes the humanization of an anti-myostatin antibody Fab by using a traditional CDR grafting approach onto clinically validated germline frameworks (Apgar *et al*., 2016). Myostatin is a member of the transforming growth factor β (TGF-β) family, regulates the skeletal muscle growth and has a well conserved biological function in all murine and human homologs with 100% sequence identity (Carnac *et al*., 2007). The third antibody series describes the humanization of the A5B7 Fab, binding to the tumor-associated glycoprotein carcinoembryonic antigen (CEA) (Banfield et al., 1996, 1997). The CEA is involved in cell adhesion and is produced in the gastrointestinal tissue. It is frequently used as a tumor marker for colorectal cancer (Hammarström, 1999). The fourth pair of antibody Fabs shows the humanization of the murine CTM01 Fab, which binds to polymorphic epithelial mucin (PEM/MUC1) (Banfield *et al*., 1997). PEM are expressed at the surfaces of human mammary cells and are involved in breast and ovarian cancer (Nath and Mukherjee, 2014; Peat *et al*., 1992). The fifth antibody series describes the humanization and the role of the Vernier zone residues of an anti-human epidermal growth factor receptor murine 528 Fv (Makabe *et al*., 2008). EGFR is a transmembrane glycoprotein with an intracellular tyrosine kinase domain and is known to play a central role in the development of tumors (Voldborg *et al*., 1997). The sixth antibody series discusses the superhumanization of the murine/wild-type anti-idiotypic Ab2/3H6 directed against the broadly neutralizing anti-HIV-1 antibody 2 F5 and represents a negative control for our simulations, because the superhumanized variant su3H6 completely lost its binding affinity (Margreitter *et al*., 2016). In Supplementary Table 1, we show an overall table of all studied humanization examples including the PDB codes, and a comparison of the antibody sequences to the mouse and human germlines for light and heavy chain respectively, using IgBlast.

## Methods

A previously published method characterizing the CDR-H3 loop ensemble in solution (Fernández-Quintero, Loeffler *et al*., 2019; Fernández-Quintero, Kraml *et al*., 2019) was used to investigate the influence of the framework on the CDR-H3 loop dynamics. We deleted the co-crystallized antigen in all complex crystal structures. A recently introduced nomenclature to distinguish between Fabs and Fvs crystallized with and without antigen is AGed and AGless, respectively, will be used in this manuscript (Fernández-Quintero, Kraml *et al*., 2019). For the humanization of the Ab2/3H6 no experimental structural information was available and therefore the initial coordinates were taken from the 3H6-2F5 crystal structure with the PDB accession code 3BQU. The superhumanized su3H6 variant was modeled using the program Molecular Operating Environment (MOE, Chemical Computing Group, version 2018.01). All starting structures for simulations were prepared in MOE using the Protonate3D tool (Labute, 2009; Molecular Operating Environment (MOE), 2018). To neutralize the charges, we used the uniform background charge (Case, Betz *et al*., 2016; Roe and Cheatham, 2013; Hub *et al*., 2014). Using the tleap tool of the AmberTools16 (Case, Betz *et al*., 2016; Roe and Cheatham, 2013) package, the crystal structures were soaked with cubic water boxes of TIP3P water molecules with a minimum wall distance of 10 Å to the protein (Jorgensen *et al*., 1983). For all crystal structures parameters of the AMBER force field 14SB were used (Maier *et al*., 2015). The antibodies were carefully equilibrated using a multistep equilibration protocol (Wallnoefer *et al*., 2011).

### Metadynamics simulations

To enhance the sampling of the conformational space well-tempered metadyamics (Barducci *et al*., 2008; Biswas *et al*., 2018; Barducci *et al*., 2011) simulations were performed in GROMACS (Abraham *et al*., 2015; Pronk *et al*., 2013) with the PLUMED 2 implementation (Tribello *et al*., 2014). As collective variables, we used a linear combination of sine and cosine of the ψ torsion angles of CDR-H3 and CDR-L3 loop calculated with functions MATHEVAL and COMBINE implemented in PLUMED 2 (Tribello *et al*., 2014). As discussed previously, the ψ torsion angle captures conformational transitions comprehensively (Ramachandran *et al*., 1963). The decision to include the CDR-L3 loop ψ torsion angles is based on the structural correlation of the CDR-L3 and CDR-H3 loop and the observed improved sampling efficiency (James and Tawfik, 2005). The simulations were performed at 300 K in an NpT ensemble. The height of the Gaussian was determined according to minimal distortion of the antibody systems, resulting in a Gaussian height of 10.0 kcal/mol. Gaussian deposition occurred every 1000 steps and a biasfactor of 10 was used. One microsecond metadynamics simulations were performed for each available antibody crystal structure. The resulting trajectories were clustered in cpptraj (Roe and Cheatham, 2013; Shao *et al*., 2007) by using the average linkage hierarchical clustering algorithm with a distance cutoff criterion of 1.2 Å resulting in a large number of clusters. The cluster representatives for the systems were equilibrated and simulated for 100 ns using the AMBER16 (Case, Betz *et al*., 2016) simulation package.

### Molecular dynamics simulations

Molecular dynamics simulations were performed in an NpT ensemble using pmemd.cuda (Salomon-Ferrer *et al*., 2013). Bonds involving hydrogen atoms were restrained by applying the SHAKE algorithm (Miyamoto and Kollman, 1992), allowing a time step of 2.0 fs. Atmospheric pressure of the system was preserved by weak coupling to an external bath using the Berendsen algorithm (Berendsen *et al*., 1984). The Langevin thermostat (Adelman and Doll, 1976) was used to maintain the temperature during simulations at 300 K.

For the obtained trajectories a time-lagged independent component analysis (tICA) using the python library PyEMMA 2 employing a lag time of 10 ns was performed (Scherer *et al*., 2015). Thermodynamics and kinetics were calculated with a Markov-state model (Chodera and Noé, 2014) by using PyEMMA 2, which uses the k-means clustering algorithm (Likas *et al*., 2003) to define microstates and the PCCA+ clustering algorithm (Röblitz and Weber, 2013) to coarse grain the microstates to macrostates. PCCA+ is a spectral clustering method, which discretizes the sampled conformational space based on the eigenvectors of the transition matrix. The sampling efficiency and the reliability of the Markov-state model (e.g. defining optimal feature mappings) can be evaluated with the Chapman–Kolmogorov test (Karush, 1961; Miroshin, 2016), by using the variational approach for Markov processes (Wu and Noé, 2017) and by taking into account the fraction of states used, as the network states must be fully connected to calculate probabilities of transitions and the relative equilibrium probabilities. To build the Markov-state model, the features and the lag time were chosen for each humanization series individually and are described for each antibody, respectively. The lag times are chosen according to the implied timescales plot at which the computed relaxation timescales are constant (Supplementary Figure S1) (Swope *et al*., 2004).

### ABangle

ABangle (Dunbar *et al*., 2013) is a computational tool to characterize the relative orientations between the antibody variable domains (V_H_ and V_L_) using six measurements (five angles and one distance of the two domains). A plane is projected on each of the two variable domains based on a core set of structurally highly conserved residues. Between those two planes a distance vector C is defined. The six measures are then two tilt angles between each plane and the distance vector (HC1, HC2, LC1 and LC2), a torsion angle between the two planes along the distance vector (HL) and the length of the distance vector (dc). The ABangle script can calculate these measures for an arbitrary Fv region by aligning a consensus structure to the found core set positions and fitting the planes and distance vector from this alignment. This tool available online was combined with an in-house python script to reduce computational time and to visualize our simulation data over time. The in-house script makes use of ANARCI (Dunbar and Deane, 2016) for fast local annotation of the Fv region and pytraj (Case, Nguyễn *et al*., 2016) for rapid trajectory processing. In the background, relative domain orientations observed in a representative dataset of the PDB are displayed.

## Results

### Humanization of anti-human IL-13 antibody

**Fig. 1:**
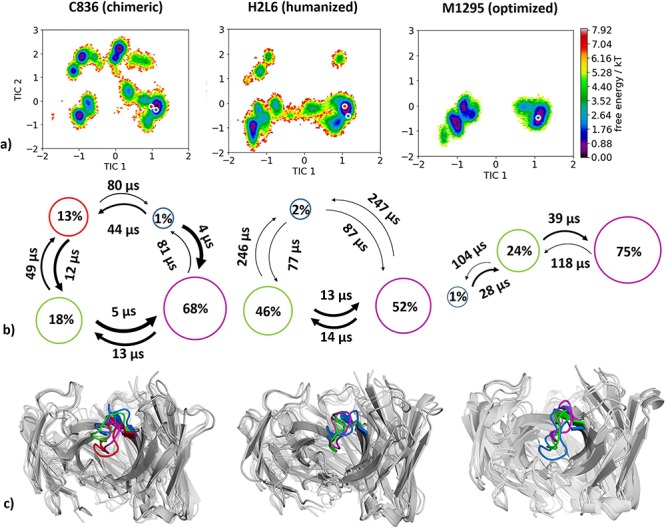
(**a**) Combined tICA plots of all humanization variants using the same tICA coordinate system with the projected X-ray structures. AGless crystal structures are colored red, while AGed crystal structures are colored blue. For the chimeric antibody we obtained 8.6 μs, for the humanized antibody 7.6 μs and for the M1295 6.1 μs of molecular dynamics trajectories. (**b**) Markov-state models with the respective state probabilities and timescales. (**c**) Representative macrostate structures are shown and color-coded according to the state probabilities in (**b**).

Five crystal structures in different stages of humanization and with and without antigen crystallized were available to study the conformational diversity of the CDR-H3 loop in different stages of humanization. The PDB accession codes for the chimeric variant crystallized with and without IL-13 are 3L5W and 3L7E, respectively. 3L7X (AGed) and 3L7F (AGless) are the PDB accession codes for the humanized variant H2L6. After the specificity-determining residue optimization the best variant is the AGed M1295 Fab with the accession code 4PS4. As described in the Methods section, we clustered the metadynamics simulations and used the resulting cluster representatives as starting structures for short molecular dynamics simulations. For the chimeric antibody, we obtained 8.6 μs, for the humanized antibody 7.6 μs and for the M1295 6.1 μs of molecular dynamics trajectories. The resulting conformational space is displayed in Fig. 1a, which directly compares all humanization variants in the same tICA coordinate space. As features to construct the tICA, we used the backbone torsions of the CDR-H3 loop and for the Markov-state model we applied a lag time of 20 ns. We clearly observe a decrease in conformational diversity of the CDR-H3 loop, combined with a substantial population shift toward the humanized and optimized antibody. Figure 1b shows the state probabilities with the respective transition timescales in the nano-to-milli-second timescale. The observed population shifts in the free energy landscapes are reflected in the resulting probabilities. The respective macrostate representatives of all humanization variants are displayed in Fig. 1c and color-coded according to the Fig. 1b.

### Humanization of anti-myostatin antibody

**Fig. 2:**
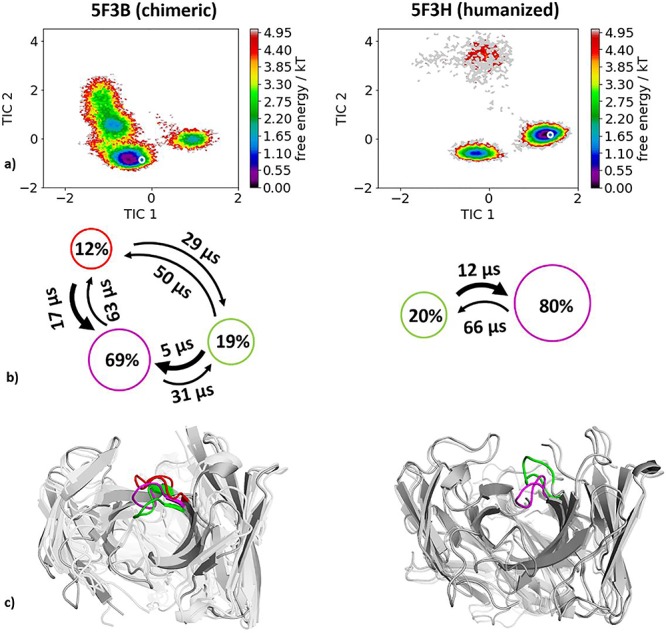
(**a**) Combined tICA plots of all humanization variants using the same tICA coordinate system with the projected X-ray structures. AGed crystal structures are colored blue. For the chimeric antibody we obtained 4.2 μs, for the humanized antibody 2.9 μs of molecular dynamics trajectories. (**b**) Markov-state models with the respective state probabilities and timescales. (**c**) Representative macrostate structures are shown color-coded according to the state probabilities in (**b**).

For the humanization of an anti-myostatin antibody experimental structural information was available in the chimeric (PDB code 5F3B) and the humanized variant (PDB code 5F3H) crystallized with myostatin present. Following the same procedure as described in the Methods section, we obtained for the chimeric variant 4.2 μs and for the humanized variant 2.9 μs molecular dynamics trajectories. As features to construct the tICA, we used the backbone torsions of the CDR-H3 loop and for the Markov-state model we applied a lag time of 10 ns. The resulting free energy landscapes with the respective state probabilities and transition timescales are shown in Fig. 2. Figure 2a compares the free energy surfaces in the same coordinate system of the chimeric and humanized antibody Fab and in line with the results in Fig. 1 we observe a population shift toward the humanized bound crystal structure. The global minimum in solution of the chimeric antibody Fab CDR-H3 loop becomes a local side-minimum. The resulting state probabilities in Fig. 2b reflect this observed population shift.

### Humanization of anti-tumor associated glycoprotein CEA antibody

**Fig. 3:**
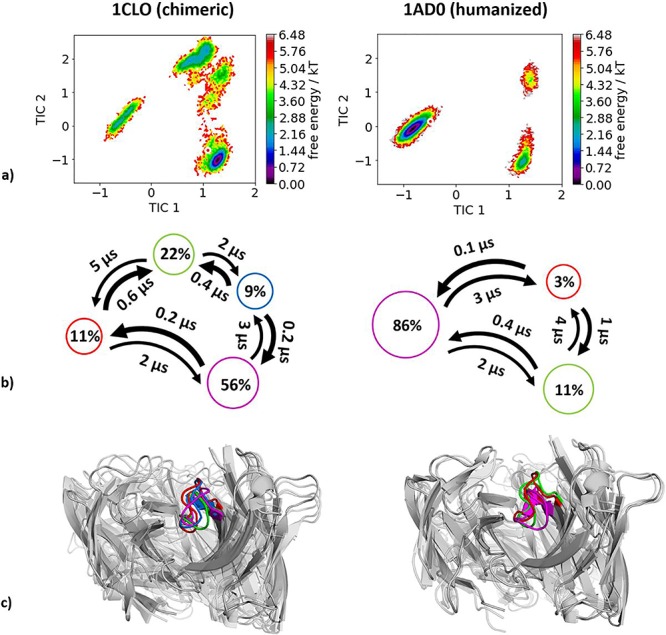
(**a**) Combined tICA plots of all available humanization variants using the same tICA coordinate system with the projected X-ray structures. AGless crystal structures are colored red. For the chimeric antibody we obtained 4.0 μs, for the humanized antibody 3.7 μs of molecular dynamics trajectories. (**b**) Markov-state models with the respective state probabilities and timescales. (**c**) Representative macrostate structures are shown and color-coded according to the state probabilities in (**b**).

Again, for the chimeric (PDB code 1CLO) and humanized (PDB code 1 AD0) variants, crystallized without antigen, experimental structural information was available and used as starting point for molecular dynamics simulations. Following the same procedure as described in the Methods section, we obtained for the chimeric variant 4.0 μs molecular dynamics trajectories and for the humanized variant 3.7 μs molecular dynamics trajectories. As features to construct the tICA, we used the backbone torsions of the CDR-H3 loop and for the Markov-state model we applied a lag time of 10 ns. Besides the substantial decrease in conformational space (Fig. 3a and b), we observe a strong conformational shift of the CDR-H3 loop conformation in solution upon humanization. This significant population shift is also reflected in the resulting state probabilities. Again, Fig. 3c shows the macrostate representative structures.

### Humanization of anti-PEM MUC1 antibody

**Fig. 4:**
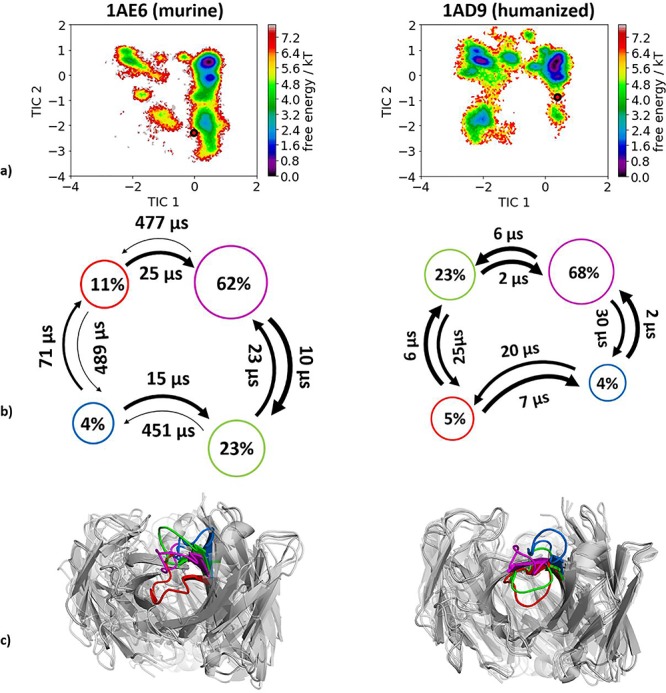
(**a**) Combined tICA plots of all humanization variants using the same tICA coordinate system with the projected X-ray structures. AGless crystal structures are colored red. For the murine antibody we obtained 17.5 μs and for the humanized antibody 13.0 μs of molecular dynamics trajectories. (**b**) Markov-state models with the respective state probabilities and timescales. (**c**) Representative macrostate structures are shown and color-coded according to the state probabilities in (**b**).

Experimental structural information was available of the murine (1AE6) and the humanized (1 AD9) anti-PEM antibody Fab. Following the same procedure as described in the Methods section, we obtained for the mouse antibody 17.5 μs molecular dynamics trajectories and for the humanized variant 13.0 μs molecular dynamics trajectories. As features to construct the tICA, we used the backbone torsions of the CDR-H3 loop and, for the Markov-state model, we applied a lag time of 10 ns. Upon humanization we observe, in line with the previous examples, a conformational shift of the CDR-H3 loop conformation in solution (Fig. 4). In this antibody humanization example, the starting crystal structures lie in very unfavorable regions and this can be explained due to crystal contacts of a symmetry mate Fab in the packing unit (Supplementary Figure S2).

### Humanization of anti-human epidermal growth factor receptor antibody

**Fig. 5:**
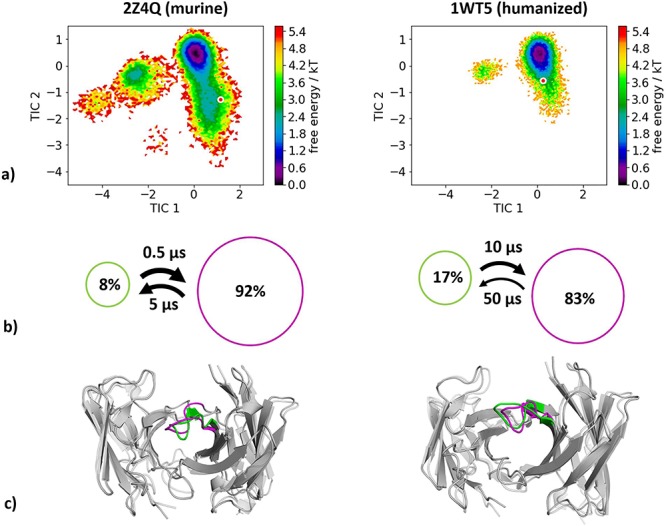
(**a**) Combined tICA plots of all available humanization variants using the same tICA coordinate system with the projected X-ray structures. AGless crystal structures are colored red. For the murine antibody we obtained 4.2 μs and for the humanized antibody 3.3 μs of molecular dynamics trajectories. (**b**) Markov-state models with the respective state probabilities and timescales. (**c**) Representative macrostate structures color-coded according to the state probabilities in (**b**).

For the last analyzed humanization pair experimental structural information of a murine (PDB accession code 2Z4Q) and a humanized (PDB accession code 1WT5) antibody variable fragment (Fv) crystallized without antigen was available. Figure 5 shows the resulting free energy surface of the CDR-H3 loop with reconstructed kinetics of 4.2 μs (murine) and 3.3 μs (humanized) of molecular dynamics trajectories. As features to construct the tICA we used the Cα-atoms of the CDR-H3 loop and for the Markov-state model we applied a lag time of 30 ns. Figure 5a reveals a substantial decrease in conformational space and shows one order of magnitude higher timescales in the humanized antibody Fv compared to the murine Fv.

### Humanization of the anti-idiotypic antibody Ab2/3H6 against the anti-HIV-1 antibody 2F5

**Fig. 6:**
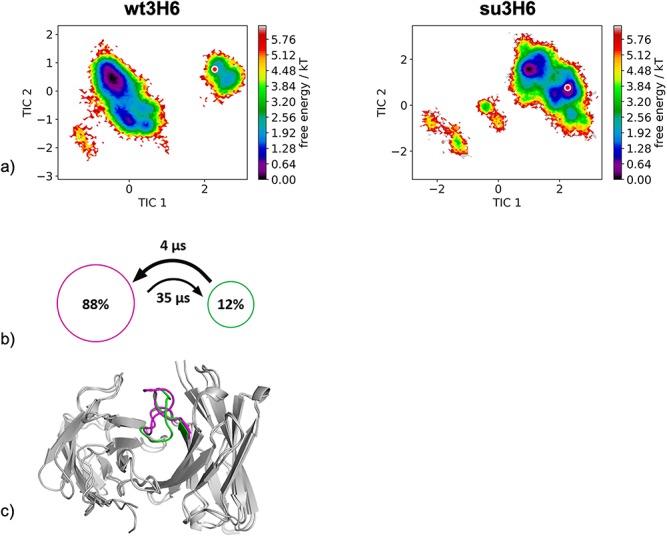
(**a**) Combined tICA plots of all available humanization variants using the same tICA coordinate system with the projected modeled starting structures. For the murine antibody we obtained 9.6 μs and for the superhumanized antibody 11.1 μs of molecular dynamics trajectories. (**b**) Markov-state models with the respective state probabilities and timescales. (**c**) Representative macrostate structures color-coded according to the state probabilities in (**b**).

As described in the Methods section, no experimental structural information was available for the wild-type 3H6 and the su3H6 variant, therefore the initial coordinates of the 3H6-2F5 complex structure (PDB accession code: 3BQU) were taken to model the starting structures for each 1 μs metadynamics simulations. Figure 6a illustrates the resulting free energy surface of the CDR-H3 loop including the transition kinetics and state probabilities of the 9.4 μs of trajectories of the murine variant and shows the 11.1 μs of trajectories of the superhumanized variant projected into the murine tICA coordinate system. To build the tICA and the Markov-state model, we applied a lag time of 10 ns according to lag time ranges in which the implied timescales are constant. We clearly see, that the murine variant reveals a kinetically different dominant CDR-H3 loop minimum in solution, compared with the superhumanized variant. The potentially binding competent CDR-H3 loop conformation of the mouse only slightly overlaps with the ensemble of the unsuccessful superhumanization variant. These results are in line with experimental binding affinity measurements, which showed that the su3H6 variant completely lost its binding ability. Besides, the Markov-state model in Fig. 6b of the wt3H6 results in two macrostates, within we identified the dominant CDR-H3 loop conformation in solution. No Markov-state model of the su3H6 variant was built in the same coordinate system, because of the distinct movements and configurations sampled. The resulting CDR-H3 loop macrostate representatives of the murine variant are illustrated in Fig. 6c.

## Discussion

In this study, we investigate the influence of the framework in the humanization process on the resulting CDR-H3 loop ensemble in solution. Together with experimental structural information in distinct stages of humanization, we elucidate in five series of antibodies differences in conformational diversity of the CDR-H3 loop and characterize kinetic and thermodynamic properties, i.e. population shifts during antibody humanization. Understanding the unique CDR-H3 loop structural and dynamic characteristics is the key challenge for the *in silico* development of antibody biotherapeutics. Proper characterization of the CDR-H3 loop is crucial to elucidate the antigen binding process. Already Pauling and Landsteiner and later on Milstein and Foote realized that the ability of the same antibody to adopt various conformations has an impact on their binding properties and their function, which can increase the effective size of the antibody repertoire (James and Tawfik, 2003; Pauling, 1940; Foote and Milstein, 1994). The idea of having an ensemble of pre-existing conformations out of which the functional ones are selected was proposed by Pauling (Pauling, 1940; James *et al*., 2003) and demonstrated by Milstein and Wedemayr (Foote and Milstein, 1994; Wedemayer *et al*., 1997). Especially when humanizing antibodies the understanding of antigen recognition and the influence of the human framework on the CDR loop dynamics, especially the CDR-H3 loop, is crucial to minimize the tendency of immunogenic responses to non-human derived antibodies (Hwang and Foote, 2005). Various approaches have been developed up to now to improve specificity, shape complementarity, affinity and reduce immunogenicity. This broad scope and importance in the treatment of human diseases make the elucidation of humanization critical in development of biotherapeutics (Safdari *et al*., 2013).

### Anti-human IL-13 antibody

**Fig. 7:**
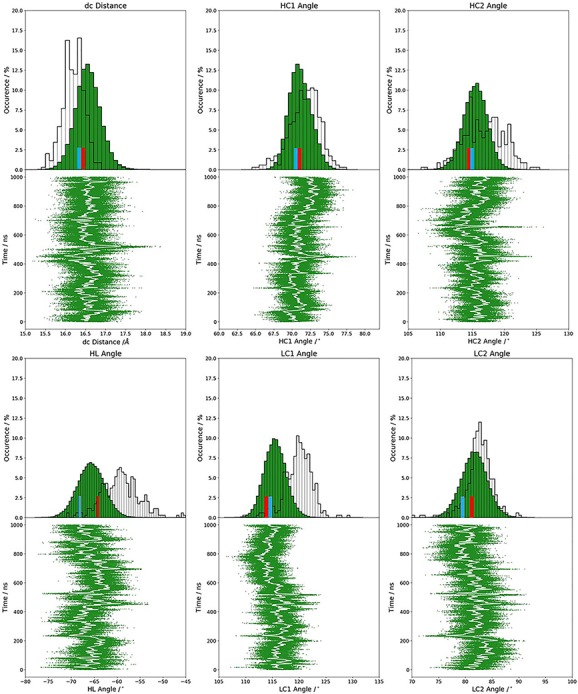
Relative V_H_-V_L_ interdomain orientations observed in 1 μs metadynamics simulation of the AGless C836 variant. The crystal structures of the 3L7E and the 3L5W are colored in red and blue, respectively.

The humanization series of the anti-human IL-13 antibody combined with strong experimental structural information in different stages of humanization crystallized with and without IL-13 offers the possibility to investigate the binding mechanism and the influence of the human framework on the CDR-H3 loop dynamics. The chimeric humanization variant C836, crystallized with and without antigen, has been discussed to indicate antigen recognition through rigid-body rotation of the V_H_-V_L_ domains (Teplyakov *et al*., 2011). Previous studies already focused on understanding and quantifying the interdomain V_H_-V_L_ orientations in antibodies (Dunbar *et al*., 2013; Bujotzek *et al*., 2015; Abhinandan and Martin, 2010; Weitzner *et al*., 2017; Marze *et al*., 2016; Fernández-Quintero *et al*., 2020). Between complex and AGless crystal structures significant variations in the relative interdomain V_H_-V_L_ orientation were reported and characterized as an induced-fit mechanism of antigen-recognition. Analyses of interdomain orientations in the obtained simulations (Fig. 7) clearly reveal conformational selection, because without the presence of the antigen the relative V_H_-V_L_ orientation was present in this pre-existing ensemble of accessible relative interdomain orientations. The influence of the framework on the CDR-H3 loop dynamics was analyzed as displayed in Fig. 1. The free energy landscape of the chimeric antibody Fab shows various deep and narrow minima, while the humanized variant reveals a shallower and broader surface. The specificity optimized humanized variant M1295 does not only show a significant decrease in conformational space but displays deep and narrow minima with transitions in the microsecond timescale.

### Humanization of anti-myostatin antibody

The humanization of the anti-myostatin antibody revealed a substantial decrease in conformational diversity and we identified a conformational shift of the dominant CDR-H3 loop conformation in solution. The free energy landscape of the chimeric antibody (Fig. 2a and b) shows that the global minimum in solution of the humanized antibody is already present as a local shallow side-minimum in this pre-existing ensemble of conformations. This result clearly reveals a strong influence of the antibody framework on the CDR-H3 loop dynamics in solution.

### Humanization of anti-tumor associated glycoprotein CEA antibody

In line with most other analyzed antibody humanizations, we observe a substantial rigidification of the CDR-H3 loop and observe that a shallow side minimum of the conformational CDR-H3 loop ensemble attached to the chimeric framework becomes the dominant conformation in solution influenced by the human framework (Fig. 3).

### Humanization of anti-PEM antibody

The humanization of the anti-tumor associated glycoprotein CEA antibody shows a conformational shift of the murine to the humanized antibody and we were able to identify an additional dominant solution conformation in the humanized variant which had been a local side-minimum in the murine antibody. In this case, we do not observe a rigidification of the CDR-H3 loop (Fig. 4a). However, especially in this case the AGless crystal structures of the antibody lie in unfavorable regions on the free energy surface. This can be explained due to crystal contacts of the tail region of the Fab symmetry mate with the CDR-H3 loop (Supplementary Figure S2). One of the two dominant CDR-H3 loop conformations in solution could be the antigen binding conformation, which could be confirmed by an X-ray structure of the complex.

### Humanization of anti-human epidermal growth factor antibody

The variable fragment of murine anti-human epidermal growth factor receptor antibody (m528Fv) has been analyzed to investigate humanization and the role of Vernier zone residues on the CDR-H3 loop dynamics. We clearly observe a significant decrease in conformational entropy (Fig. 5). Mutations in the Vernier zone clearly affect the CDR-H3 loop dynamics and shift the populations toward the global minimum in solution. Changes in conformational entropy upon humanization of murine antibodies as shown in this example have to be taken into consideration and optimized (Makabe *et al*., 2008).

### Superhumanization of the anti-idiotypic antibody Ab2/3H6 against the anti-HIV-1 antibody 2F5

The superhumanization of the murine wt3H6 antibody to the su3H6 variant represents an unsuccessful example of humanization. Even if the modeled starting structures of the metadynamics simulations were based on the same coordinates, our results clearly revealed a strong separation between the dominant CDR-H3 loop ensemble in solution of the mouse compared to the ensemble of the superhumanized antibody, which completely lost its binding affinity. This result suggests that within our obtained CDR-H3 loop ensemble in solution of the wt3H6 the potentially binding competent state of the murine variant might be present. Thus, this could again represent an example of conformational selection, because the binding competent state is present within the ensemble of pre-exisiting conformations, without the presence of the antigen. The free energy in Fig. 6a clearly shows that the starting model based on the coordinates of the 3BQU crystal structure with a 3 Å resolution does not represent the dominant CDR-H3 loop solution structure of the mouse. This observation is in line with previous studies, because structure prediction in particular of the CDR-H3 loop, due to its unique characteristics (Regep *et al*., 2017), remains challenging and therefore should be characterized as conformational ensemble in solution (Fernández-Quintero, Kraml *et al*., 2019).

Our results suggest that our simulations can be used to characterize the CDR-H3 loop ensemble upon humanization to identify potentially binding competent humanization variants, which share a similar CDR-H3 loop ensemble in solution with the murine counterparts. The observed rigidification in some cases might be a consequence of our chosen examples, because we could only find successful humanization cases with experimental structural information in which the human frameworks and point mutations were already optimized to bind the antigen with a similar affinity as the murine variant. However, for the failed humanization example, even when using a homology model as starting point, we were not only able to sample a broader CDR-H3 loop ensemble upon superhumanization but also observed nearly no overlap with the murine conformational space (Fig. 6a). Our results show that our simulations can be used to identify the binding competent CDR-H3 loop conformations even without the antigen present and characterize changes in the CDR-H3 loop ensemble and transition kinetics upon humanization.

## Conclusion

We investigated the influence of the human framework on the CDR-H3 loop dynamics and observed in all considered and successful antibody humanization variants a strong conformational shift of the dominant CDR-H3 loop conformation in solution and in most of the cases a substantial decrease in the conformational diversity of the CDR-H3 loop. We also included a failed humanization series which revealed barely any structural and kinetic overlap of the superhumanized variant with the potentially binding competent CDR-H3 loop ensemble in solution of the mouse. These results show that our simulations can be used to characterize the CDR-H3 loop ensemble in solution upon humanization and give valuable insights in the influence of the framework and point mutations on the resulting CDR-H3 loop conformational dynamics. Additionally, our dominant CDR-H3 loop solution structures are in agreement with binding competent CDR-H3 loop conformations. Thus, characterization and optimization of changes of conformational diversity upon humanization emerges as a key aspect of antibody humanization.

## Supplementary Material

SI_gzaa004

## PEDS Board Member

Ruth Nussinov.

## Notes

Paper edited by: Ruth Nussinov, Board Member for PEDS
