# Supplementary material for: Antibody humanization—the Influence of the antibody framework on the CDR-H3 loop ensemble in solution
Source: Protein Eng Des Sel. 2020 Mar 4;32(9):411–22. doi: 10.1093/protein/gzaa004 (PMC7098879; doi:10.1093/protein/gzaa004)
Supplement: SI_gzaa004 [file si_gzaa004.docx]

Supplemental Information

Table 1: Overview of all studied humanization systems and a comparison of the antibody sequences to the mouse and human germline genes, including sequence identities using IgBlast.

**Light chain**

| Antibody | PDB Code | Germline Gene | E-Value | Identity / % | Organism for Query |
| --- | --- | --- | --- | --- | --- |
|  |  |  |  |  |  |
| IL-13 (chimeric) | 3L7E | IGKV16-104*01  IGKV1-9*01 | 6e-68  3e-51 | 97.9  73.7 | Mouse  Human |
| IL-13 (humanized) | 3L7F | IGKV16-104*01  IGKV3-11*01 | 2e-56  3e-57 | 80.2  86.3 | Mouse  Human |
| IL-13 (optimized) | 4PS4 | IGKV16-104*01  IGKV3-11*01 | 3e-53  1e-55 | 78.1  90.0 | Mouse  Human |
|  |  |  |  |  |  |
| Anti-Myostatin (chimeric) | 5F3B | IGKV6-14*01  IGKV1-NL1*01 | 1e-65  5e-46 | 96.8  68.4 | Mouse  Human |
| Anti-Myostatin (humanized) | 5F3H | IGKV6-17*01  IGKV1-NL1*01 | 7e-55  1e-56 | 82.1  84.2 | Mouse  Human |
|  |  |  |  |  |  |
| A57B (murine) | 1CLO | IGKV4-72*01  IGKV3-11*01 | 3e-63  1e-38 | 93.7  62.1 | Mouse  Human |
| A57B (humanized) | 1AD0 | IGKV4-72*01  IGKV1-16*01 | 4e-49  6e-48 | 72.6  77.2 | Mouse  Human |
|  |  |  |  |  |  |
| CTM01 (murine) | 1AE6 | IGKV2-137*01  IGKV2-18*01 | 4e-69  1e-53 | 96.0  76.0 | Mouse  Human |
| CTM01 (humanized) | 1AD9 | IGKV2-137*01  IGKV1-5*03 | 3e-48  2e-51 | 67.0  77.8 | Mouse  Human |
|  |  |  |  |  |  |
| Anti-Human EGFR (murine) | 2Z4Q | IGKV1-117*01  IGKV2-29*02 | 2e-67  1e-55 | 92.0  77.0 | Mouse  Human |
| Anti-Human EGFR | 1WT5 | IGKV1-117*01  IGKV2-29*02 | 2e-64  2e-62 | 86.0  84.0 | Mouse  Human |
|  |  |  |  |  |  |
| wt3H6 |  | IGKV17-121*01  IGKV5-2*01 | 2e-68  1e-42 | 96.8  63.2 | Mouse  Human |
| su3H6 |  | IGKV17-121*01  IGKV5-2*01 | 2e-68  1e-41 | 96.8  63.2 | Mouse  Human |

**Heavy Chain**

| Antibody | PDB Code | Germline Gene | E-Value | Identity / % | Organism for Query |
| --- | --- | --- | --- | --- | --- |
|  |  |  |  |  |  |
| IL-13 (chimeric) | 3L7E | IGHV8-8*01  IGHV2-5*02 | 3e-66  9e-50 | 92.0  71.4 | Mouse  Human |
| IL-13 (humanized) | 3L7F | IGHV8-8*01  IGHV2-5*02 | 7e-56  9e-61 | 76.0  85.7 | Mouse  Human |
| IL-13 (optimized) | 4PS4 | IGHV8-8*01  IGHV2-5*02 | 2e-55  4e-61 | 75.0  86.7 | Mouse  Human |
|  |  |  |  |  |  |
| Anti-Myostatin (chimeric) | 5F3B | IGHV5-9-3*01  IGHV3-21*01 | 8e-68  2e-56 | 99.0  82.7 | Mouse  Human |
| Anti-Myostatin (humanized) | 5F3H | IGHV5-9-3*01  IGHV3-23*03 | 2e-60  7e-64 | 86.7  94.9 | Mouse  Human |
|  |  |  |  |  |  |
| A57B (murine) | 1CLO | IGHV7-3*02  IGHV3-71*01 | 1e-69  7e-57 | 96.0  81.0 | Mouse  Human |
| A57B (humanized) | 1AD0 | IGHV7-3*02  IGHV3-71*01 | 8e-64  2e-58 | 88.1  82.0 | Mouse  Human |
|  |  |  |  |  |  |
| CTM01 (murine) | 1AE6 |  | 2e-69  2e-51 | 98.0  70.4 | Mouse  Human |
| CTM01 (humanized) | 1AD9 | IGHV1-84*02  IGHV1-3*01 | 9e-60  2e-55 | 82.7  76.5 | Mouse  Human |
|  |  |  |  |  |  |
| Anti-Human EGFR (murine) | 2Z4Q | IGHV1S22*01  IGHV1-2*06 | 6e-57  1e-44 | 84.2  65.3 | Mouse  Human |
| Anti-Human EGFR | 1WT5 | IGHV1S61*01  IGHV1-2*06 | 9e-56  7e-66 | 76.5  91.8 | Mouse  Human |
|  |  |  |  |  |  |
| wt3H6 |  | IGHV1S34*01  IGHV1-3*01 | 4e-60  1e-45 | 90.9  64.9 | Mouse  Human |
| su3H6 |  | IGHV1S12*01  IGHV1-69-2*01 | 1e-50  1e-62 | 69.1  87.8 | Mouse  Human |


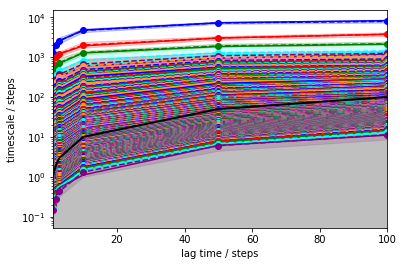


SI Figure S1: Exemplary implied timescale plot of the humanization example of the 1AE6 murine antibody to illustrate how the lag times are chosen. In this case we chose a lag time of 100 steps which corresponds to a lag time of 10 ns, because of the constant relaxation lag times after 10 ns.


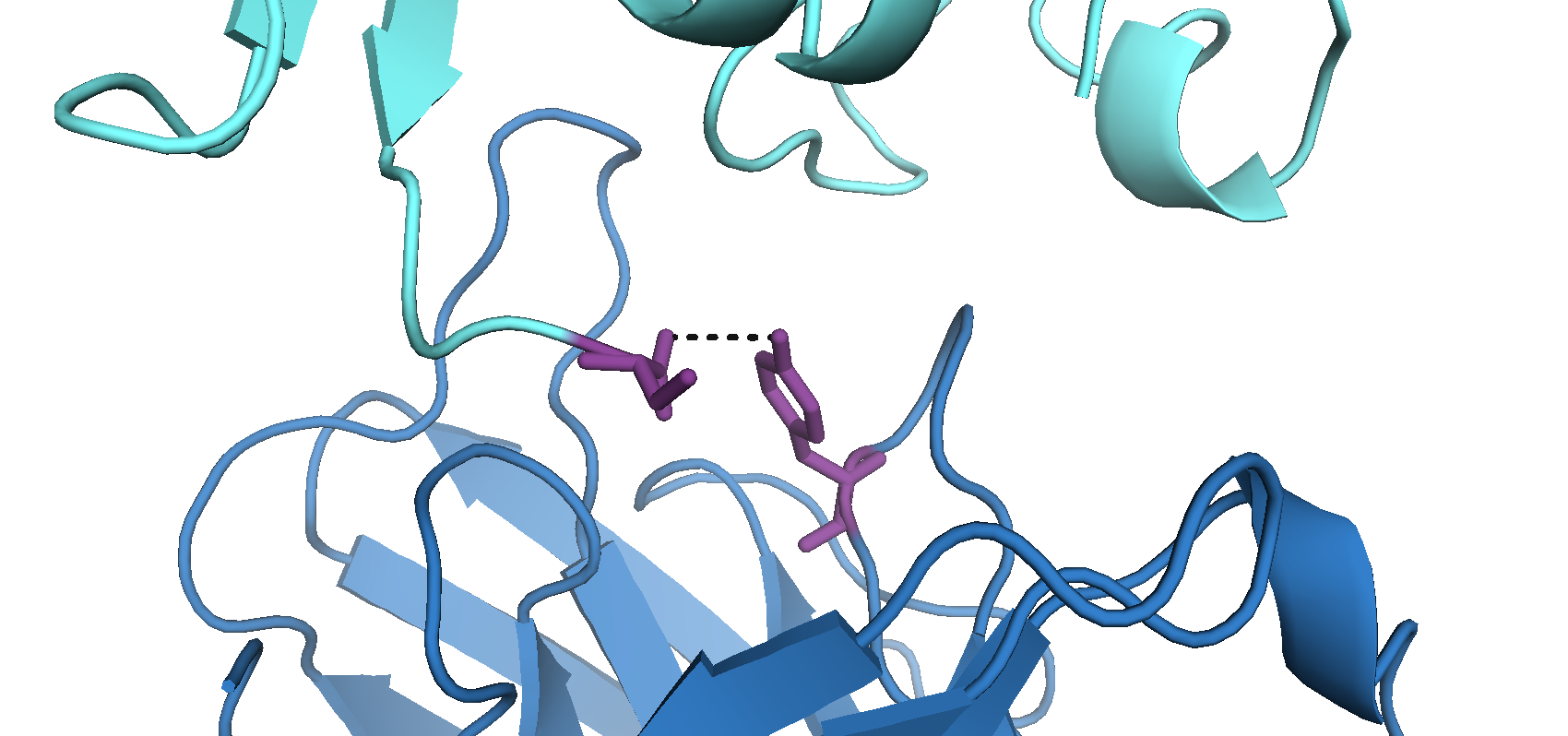


SI Figure S2: Crystal contacts of the tail region of the symmetry mate Fab with the CDR-H3 loop of the humanized anti-polymorphic epithelial mucin antibody.


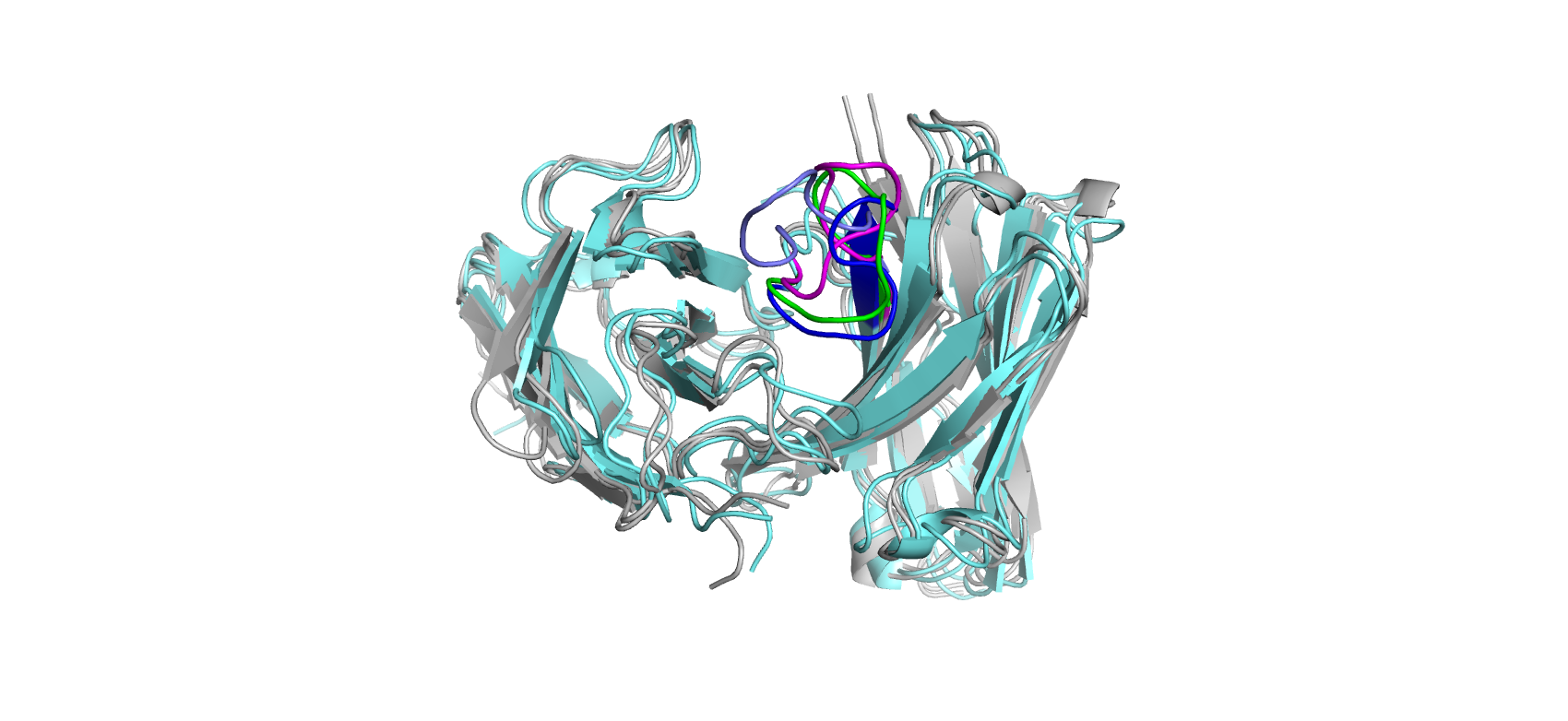


SI Figure S3: Macrostate representatives of the murine wt3H6 (grey) compared to the highest populated cluster representatives of the su3H6 variant (cyan). The CDR-H3 loop macrostate representatives are colored in green and magenta, according to Figure 6c, while the cluster representatives of the su3H6 are colored in light blue and blue.
